# Age-related macular degeneration, subretinal drusenoid deposits, and cuticular and calcified drusen in black and hispanic subjects

**DOI:** 10.1186/s40942-025-00710-4

**Published:** 2025-07-28

**Authors:** John M. Tan, Yang Fei, Liang Wang, Oscar Otero-Marquez, Tasin R. Bhuiyan, J. Fernando Arevalo, Gareth M.C. Lema, Roland Theodore Smith

**Affiliations:** 1 Department of Ophthalmology, Icahn School of Medicine at Mount Sinai, New York Eye and Ear Infirmary of Mount Sinai, New York, NY USA; 2 Department of Ophthalmology, JJ Peters VA Medical Center, Bronx, NY USA; 3 Department of Ophthalmology, Johns Hopkins School of Medicine, Baltimore, MD USA

**Keywords:** Age-related macular degeneration, Drusen, Ethnicities, Vascular disease

## Abstract

**Background:**

Subretinal drusenoid deposits (SDDs), cuticular drusen, and calcified drusen have been linked to rapid progression of age-related macular degeneration (AMD). SDDs have also been linked to high-risk vascular diseases (HRVDs). However, SDDs, cuticular drusen, and calcified drusen have not been reported in Black and Hispanic populations. We report that these drusen phenotypes occur in Black and Hispanic AMD patients.

**Methods:**

Twenty-three Black and Hispanic AMD subjects were identified in a published cross-sectional study of 200 AMD subjects. Spectral-domain optical coherence tomography, near-infrared reflectance imaging, and lipid profiles were obtained in the parent study. Masked readers assigned subjects into 2 groups: SDDs, present with or without drusen, and drusen only, as in the parent study. Calcified and cuticular drusen were independently identified. Subjects were assigned by health history questionnaires into those with or without HRVDs, defined as: cardiac valve defect (i.e., aortic stenosis), myocardial defect (i.e., myocardial infarction), and stroke/transient ischemic attack.

**Results:**

10/23 subjects were in the SDD group (3 Black and 7 Hispanic subjects), 13 of 23 were in the drusen only group. 4/23 subjects were identified with cuticular drusen (1 Black and 3 Hispanic subjects) and 4/23 subjects were identified with calcified drusen (2 Black and 2 Hispanic Subjects). All subjects had respective phenotypes indistinguishable from that of White subjects. 3/10 SDD subjects had HRVDs.

**Conclusions:**

We report, for the first time to our knowledge, that subretinal drusenoid deposits, calcified drusen, and cuticular drusen are present in some AMD patients who identify as Black or Hispanic. A strong association of SDDs with HRVDs was discovered in the parent study. These diseases are known to be over-represented in these under-served populations. SDDs, calcified drusen, and cuticular drusen also confer high risk for progression to advanced AMD. A diligent search for these drusen phenotypes in minority patients with AMD or with HRVDs is thus warranted. Further studies of larger cohorts of Black and Hispanic AMD subjects are needed to better assess associations of these drusen subtypes with life threatening diseases.

**Supplementary Information:**

The online version contains supplementary material available at 10.1186/s40942-025-00710-4.

## Background

**Fig. 1:**
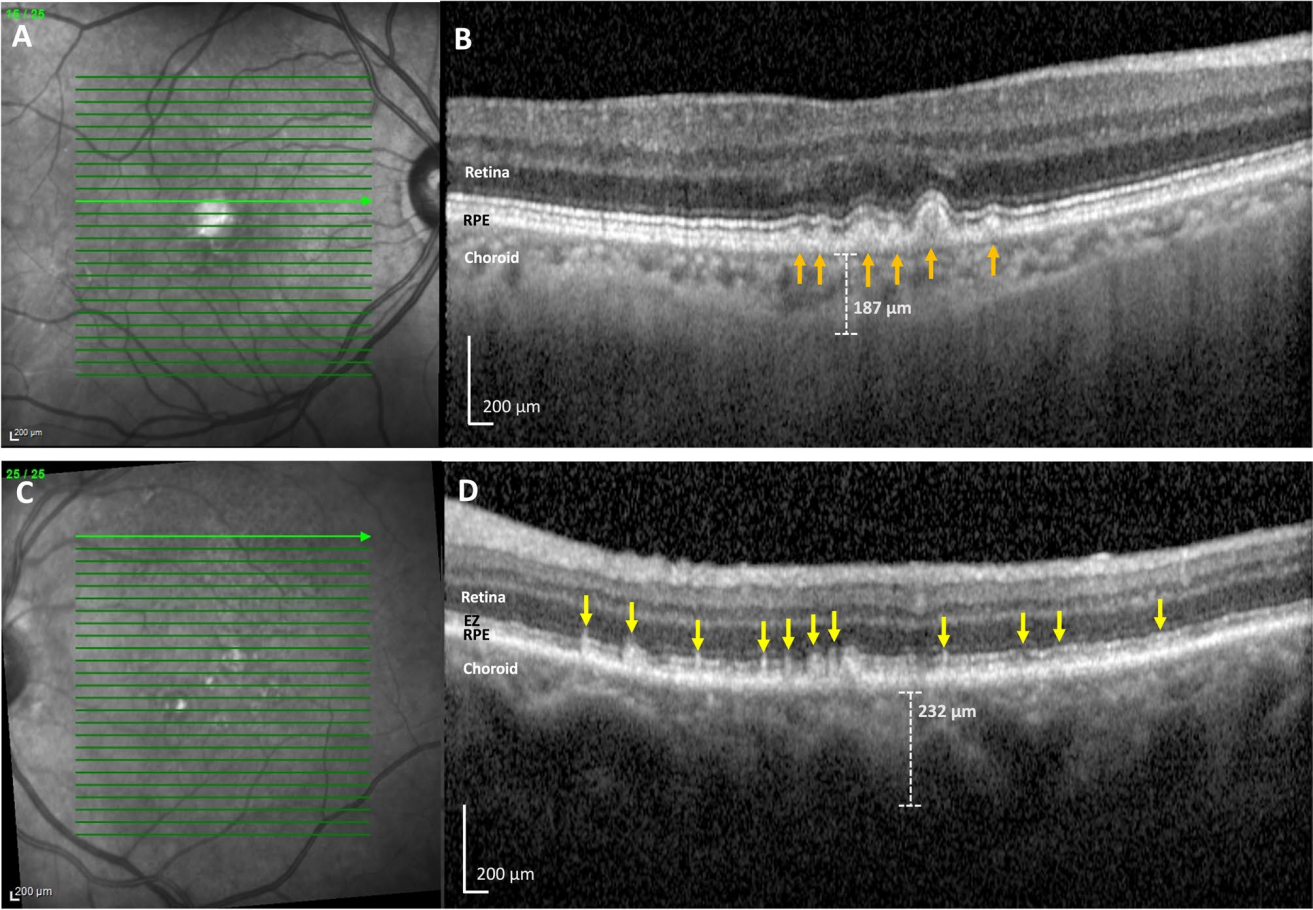
(**A-B**) Hispanic subject with soft drusen and no subretinal drusenoid deposits (SDDs), right eye. (**C-D**) Hispanic subject with SDDs and no soft drusen, left eye. (**A**) Near-infrared imaging (NIR) scan. There are hyper-reflective lesions, characteristic of soft drusen. The green line indicates the location of SD-OCT scan. (**B**) Spectral domain optical coherence tomography (SD-OCT) B-scan. The B-scan shows soft drusen lumps of intermediate reflectivity on Bruch’s membrane elevating the RPE (orange arrows). The choroidal thickness, measured from the base of the RPE to the choroidal–scleral interface is 187 μm. Subfoveal choroidal thickness, not shown, was measured at 131 μm. (**C**) Near-infrared imaging (NIR) scan. There are homogeneous and moderately hyporeflectant lesions characteristic of SDDs. The green line indicates the location of SD-OCT scan. (**D**) Spectral domain optical coherence tomography (SD-OCT) B-scan. The B-scan reveals many hyper-reflective subretinal lesions (yellow arrows), with a conical appearance above the retinal pigment epithelium (RPE), distorting (stage 2) and penetrating (stage 3) the ellipsoid zone (EZ). Choroidal thickness, measured from the base of the RPE to the choroidal–scleral interface is 232 μm. Subfoveal choroidal thickness, not shown, was measured at 111 μm

Subretinal drusenoid deposits (SDDs), also referred to as reticular pseudo drusen (RPD) [1], and soft drusen are two important extracellular deposits, amongst others, and are two main forms of intermediate age-related macular degeneration (iAMD) on spectral-domain optical coherence tomography (SD-OCT), above and below, respectively, the retinal pigment epithelium (RPE) (shown in Fig. 1) [2]. SDDs are prevalent in AMD, with reported prevalences ranging from 9 to 70% in patients with AMD [3]. In the most precise study [4], SDD prevalence was 23% in subjects without AMD and 52% in AMD subjects (*p* < 0.0001) by the standard drusen definition of AMD, thus also suggesting that SDDs should be considered a subtype of AMD. While not specific to AMD or iAMD, SDDs have also been linked to more rapid progression of AMD than drusen only AMD [5], and are associated with high-risk vascular diseases (HRVDs) such as myocardial infarction (MI), congestive heart failure (CHF), cardiac valvular disease, and stroke/transient ischemic attack (TIA) [6]. 

On SD-OCT, cuticular drusen are small elevations of the RPE in the sub-RPE-basal laminar compartment that can present with a saw-tooth appearance, hyporeflective internal contents, and a hyperreflective rim [7]. Calcified drusen, also referred to as refractile drusen, are characterized by hyperreflective caps with hyperreflective dots that overly a hyporeflective core of calcified nodules on SD-OCT [8]. Comparatively, on SD-OCT, soft drusen are larger dome-like deposits under the RPE and SDDs are accumulations above the RPE [9]. While cuticular drusen are not associated with progression to late AMD [10], calcified drusen are [11], and both drusen phenotypes are linked to various features of advanced AMD including macular neovascularization and geographic atrophy [11–13]. 

Despite the high risks associated with SDDs, cuticular drusen, and calcified drusen, none have been reported, to our knowledge, in Hispanics or Blacks. We have accordingly performed a post hoc study of a published cross-sectional study of iAMD to test our hypothesis: SDDs, cuticular drusen, and calcified drusen are present among Hispanic and Black patients with AMD.

To our knowledge, in fact, East Asians are the only non-White group in which SDDs, cuticular drusen, or calcified drusen are reported [14, 15]. This might suggest, without data, that the prevalence of SDDs, cuticular drusen, and calcified drusen in Hispanics and Blacks is simply too low to warrant further investigation. However, although Black and Hispanic populations are known to have lower prevalences of AMD in general compared to Caucasian populations, they are still substantial, 3.62% and 3.81% for Blacks and Hispanics respectively compared to 5.40% for Caucasians [16]. This suggests the same may be true for SDDs, cuticular drusen, and calcified drusen. We therefore further suggest that SDDs, cuticular drusen, and calcified drusen among Black and Hispanic populations deserve significant further investigation.

This study is the first to our knowledge to report the SDD, cuticular drusen, and calcified drusen phenotypes in Blacks and Hispanics with AMD. Importantly, this is not a large population study for statistical analyses of SDD, cuticular drusen, and calcified drusen prevalence and association with HRVDs in Black and Hispanic populations. However, we report systemic and metabolic findings relevant to AMD and HRVDs, and describe the phenotypic, morphologic, and distributional characteristics of SDDs, cuticular drusen, and calcified drusen in our Black and Hispanic subjects for completeness.

## Methods

This current *post hoc* study was drawn from the larger parent cross-sectional study (*n* = 200) that compared risk factors in patients from two AMD cohorts (patients with SDDs, with or without drusen, and patients who only had drusen) [6]. All imaging data was previously acquired in the published parent study, and all details regarding data acquisition, imaging, and classification of drusen and SDDs can be found in the parent study and the supplemental file [6]. Other risks are also detailed in the parent study and supplemental file, such as cardiovascular risk profiling and smoking history, and are used therein to generate the reported, multivariate corrected, associations of HRVDs and SDDs, and are not repeated here. Informed consent was obtained per the parent study. The institutional review board (IRB) of Mount Sinai School of Medicine approved the study, IRB approval no. 19–00437, which adhered to the tenets of the Declaration of Helsinki.

The AMD cohorts included subjects from two tertiary vitreoretinal referral centers in New York City, New York, USA. Data was collected from August 2019 to November 2021. A 14 month break occurred due to the Covid-19 pandemic. A patient questionnaire assessed age, gender, and self-reported race/ethnicity (White, Black, Hispanic, Asian). It also covered basic medical, eye and vascular disease history, medications, and known social and clinical risks for HRVDs and AMD, including smoking.

### Inclusion and exclusion criteria

Only patients who identified as Black (*n* = 5) or Hispanic (*n* = 18) were included in this study, 23 total (11.5% of the total parent study cohort). Patients in the original study that identified as White or Asian were not included. All patients were part of the original study and had a diagnosis of iAMD on SD-OCT in at least one eye. Typically, iAMD has been defined by soft drusen and/or SDDs on SD-OCT [2, 17, 18], which is the definition used for the parent study and this study. Patients younger than 50 years of age were excluded in order to only include patients with age related rather than genetic forms of macular degeneration. Neither family history nor clinical exam of patients in our study suggested other less common genetic or retinal degenerations with SDDs. Patients with other retinal degenerations and retinal vascular diseases (i.e., diabetic retinopathy), prior retinal surgery, and/or inconclusive diagnoses were excluded. Patients with bilateral advanced AMD were also excluded.

### HRVD designation

Vascular history was collected from all patients through questionnaires and then confirmed on electronic medical records. The designation of HRVD was given to subjects who had reported myocardial defects (i.e., MI, CHF, coronary artery bypass graft (CABG), cardiomyopathy), cardiac valve defects, or stroke/TIA. These designations were deemed as likely causes of decreased systemic or ocular perfusion by experts in the field from the original study. Subjects who did not fall under these designations were categorized as non-HRVD.

### Imaging and drusen phenotype classification

Volume spectral domain optical coherence tomography (SD-OCT) scans (27 lines, automated retinal tracking, 16 scans averaged per line, good quality at least (29–34) per the device specifications), and near-infrared reflectance (NIR) scans (both 30°) centered on the macula, were obtained in the parent study on the Heidelberg Spectralis HRA + OCT (Heidelberg Engineering, Heidelberg, Delaware, USA). All scans were done with the proprietary automated retinal tracking from Heidelberg Spectralis which achieves sufficient signal to noise ratio to attain 1-micron lateral resolution on SD-OCT scans. All SD-OCT images were taken by certified retinal angiographers who are experienced experts in OCT image acquisition and centered images on the macula as defined by standard protocols based on retinal imaging fiducials. Using published protocols [7, 8, 19, 20], SD-OCT images were analyzed and identified by experts in the field for SDDs, cuticular drusen, calcified drusen, and soft drusen. NIR images were used to confirm these findings. Subjects were then designated to two groups as in the parent study, SDDs with or without drusen, and only drusen. Post-hoc analysis was done to identify cuticular drusen and calcified drusen within these groups. Cuticular drusen are characterized as small elevations of the RPE-basal laminar band that can present with a saw-tooth appearance and hyporeflective internal contents on SD-OCT [7]. Calcified drusen are characterized by hyperreflective caps and heterogenous internal reflectivity, with hyperreflective calcified nodules that overly a hyporeflective core on SD-OCT [8]. Choroidal thickness was measured in the sub-foveal region on central SD-OCT images with the caliper provided on the Heidelberg Explorer software.

### Serum risks

In the parent study, subjects had blood samples taken for HRVD risk biomarkers [21] (high-density lipoprotein (HDL), low-density lipoprotein (LDL), and triglycerides). These samples had been analyzed by spectrophotometry at Quest Diagnostics, Teterboro, New Jersey, USA.

### Statistical analysis

No statistical comparisons were made between groups or across subjects in this study and other studies. Any values presented between groups should be used as a reference, but not as statistically significant. Mean values and standard deviations (SD) are presented for numerical variables. Prevalences of study risks (risks from the questionnaire) and study outcomes (lesion subtypes) in our study are also presented.

## Results

### Demographic and clinical characteristics

There were a total of 23 subjects who identified as Black or Hispanic (Table 1). Overall, 10 subjects were in the SDDs with or without drusen group (43.5%, 10/23 total subjects, 3 Black and 7 Hispanic subjects) and 13 (56.5%, 13/23 total subjects, 2 Black and 11 Hispanic subjects) were in the drusen only group. 4 subjects had cuticular drusen (17.4%, 4/23 total subjects, 1 Black and 3 Hispanic subjects) and 4 subjects had calcified drusen (17.4%, 4/23 total subjects, 2 Black and 2 Hispanic subjects). Subjects with cuticular and calcified drusen occurred in both the SDD and drusen only groups.

**Table 1:** Demographic and Clinical Characteristics

	**Black (% of all Black subjects)**	**Hispanic (% of all Hispanic subjects)**	**Total**/*Average***(% of all subjects)**
Total Subjects	5	18	**23**
Males	3 (60.0%)	7 (38.9%)	**10 (43.5%)**
Females	2 (40.0%)	11 (61.1%)	**13 (56.5%)**
History of Smoking > 6months	1 (20.0%)	8 (44.4%)	**9 (39.1%)**
History of Hypertension	5 (100%)	12 (66.7%)	**17 (73.9%)**
Average Age (years)	75.0	78.3	*77.6*
Subjects with SDDs	3 (60.0%)	7 (38.9%)	**10 (43.5%)**
Subjects with HRVDs	1 (33.3% of Black SDD subjects)	2 (28.6% of Hispanic SDD subjects)	**3 (30.0% of all SDD subjects)**
Average Age (years)	76.7	78.9	*78.2*
Subjects with Cuticular Drusen	1 (20.0%)	3 (16.7%)	**4 (17.4%)**
Average Age (years)	90.0	88.7	*89.0*
Subjects with Calcified Drusen	2 (40.0%)	2 (11.1%)	**4 (17.4%)**
Average Age (years)	76.5	67.5	*72.0*

In SDD subjects, sub-foveal choroidal thickness (sfCTh) was measurable in 16/20 eyes, and in all subjects without SDDs (26 eyes). Mean sfCTh was lower in SDD subjects, 166.6 μm (SD 59.2 μm), than in subjects without SDDs, 208.5 μm (SD 83.2 μm). Right and left eyes were tested separately. Lower choroidal thickness is not associated with cuticular or calcified drusen in literature and thus was not analyzed directly in cuticular or calcified drusen subjects.

Three of the 10 SDD (30%) subjects had HRVDs (1 Black subject and 2 Hispanic subjects). In subjects with SDDs vs. subjects with just soft drusen, mean HDL serum levels were lower, 53.0 mg/dL (SD 12.5 mg/dL) vs. 64.5 mg/dL (SD 27.6 mg/dL) in Black subjects and 51.0 mg/dL (SD 11.9 mg/dL) vs. 59.5 mg/dL (SD 14.6 mg/dL) in Hispanic subjects. Mean LDL levels and triglyceride levels are not associated with SDDs in literature and are thus not analyzed further [22], but are reported for completeness as follows. In subjects with SDDs vs. subjects with just soft drusen, mean LDL serum levels were 116.3 mg/dL (SD 47.4 mg/dL) vs. 72.5 mg/dL (SD 37.5 mg/dL) in Black subjects and 88.6 mg/dL (SD 52.2 mg/dL) vs. 93.1 mg/dL (SD 24.6 mg/dL) in Hispanic subjects. In subjects with SDDs vs. subjects with just soft drusen, mean triglyceride serum levels were 146.7 mg/dL (SD 97.9 mg/dL) vs. 119.0 mg/dL (SD 19.8 mg/dL) in Black subjects and 126.8 mg/dL (SD 50.8 mg/dL) vs. 162.5 mg/dL (SD 65.4 mg/dL) in Hispanic subjects.

### Subject imaging examples

**Fig. 2:**
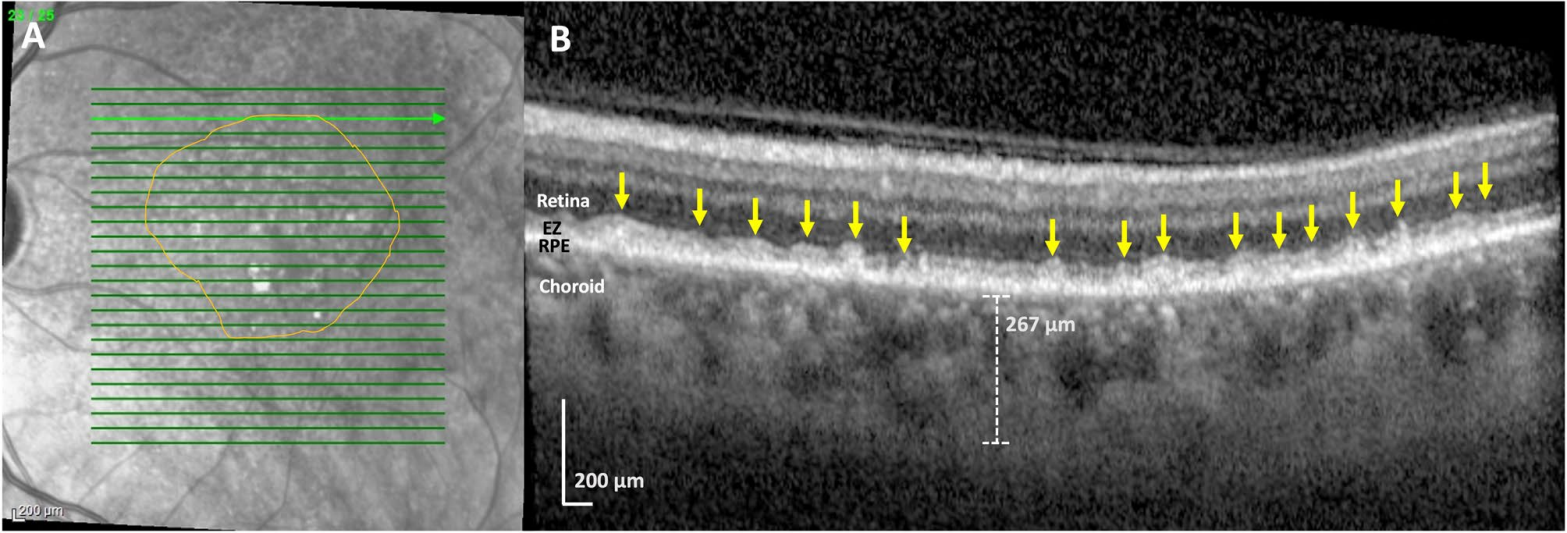
Black subject with heart failure, subretinal drusenoid deposits (SDDs), and drusen, left eye. (**A**) Near-infrared reflectance (NIR) scan. There are both hypo and hyper-reflective lesions, typical of SDDs and drusen, respectively. SDDs are present throughout, and drusen are present centrally (orange circle). The green line indicates location of the SD-OCT scan, which passes through SDDs and superiorly to the drusen. (**B**) Spectral domain optical coherence tomography (SD-OCT) B-scan. The B-scan reveals multiple confluent hyper-reflective lesions between the RPE and ellipsoid zone (EZ) and also penetrating the EZ (yellow arrows), indicative of SDDs. There are no visible drusen on the B-scan. Choroidal thickness is 267 μm. Subfoveal choroidal thickness, not shown, was measured at 106 μm, significantly thinner than for age-matched normals

**Fig. 3:**
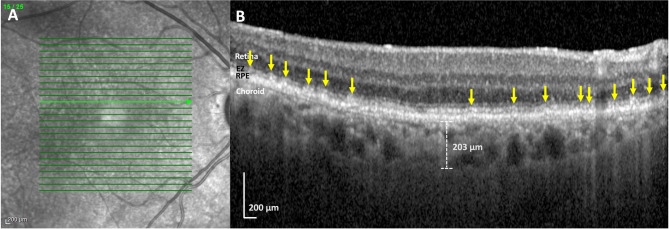
Hispanic subject with aortic regurgitation and subretinal drusenoid deposits (SDDs),right eye. (**A**) Near-infrared reflectance (NIR) scan. There are homogenous hyporeflectant lesions, typical of SDDs. The green line indicates the location of SD-OCT scan. (**B**) Spectral domain optical coherence tomography (SD-OCT) B-scan. The B-scan reveals many discrete hyper-reflective subretinal lesions in a ‘dots’ pattern (yellow arrows). There are no visible drusen. Choroidal thickness was 203 µm. Subfoveal choroidal thickness, not shown, was measured at 193 µm

**Fig. 4:**
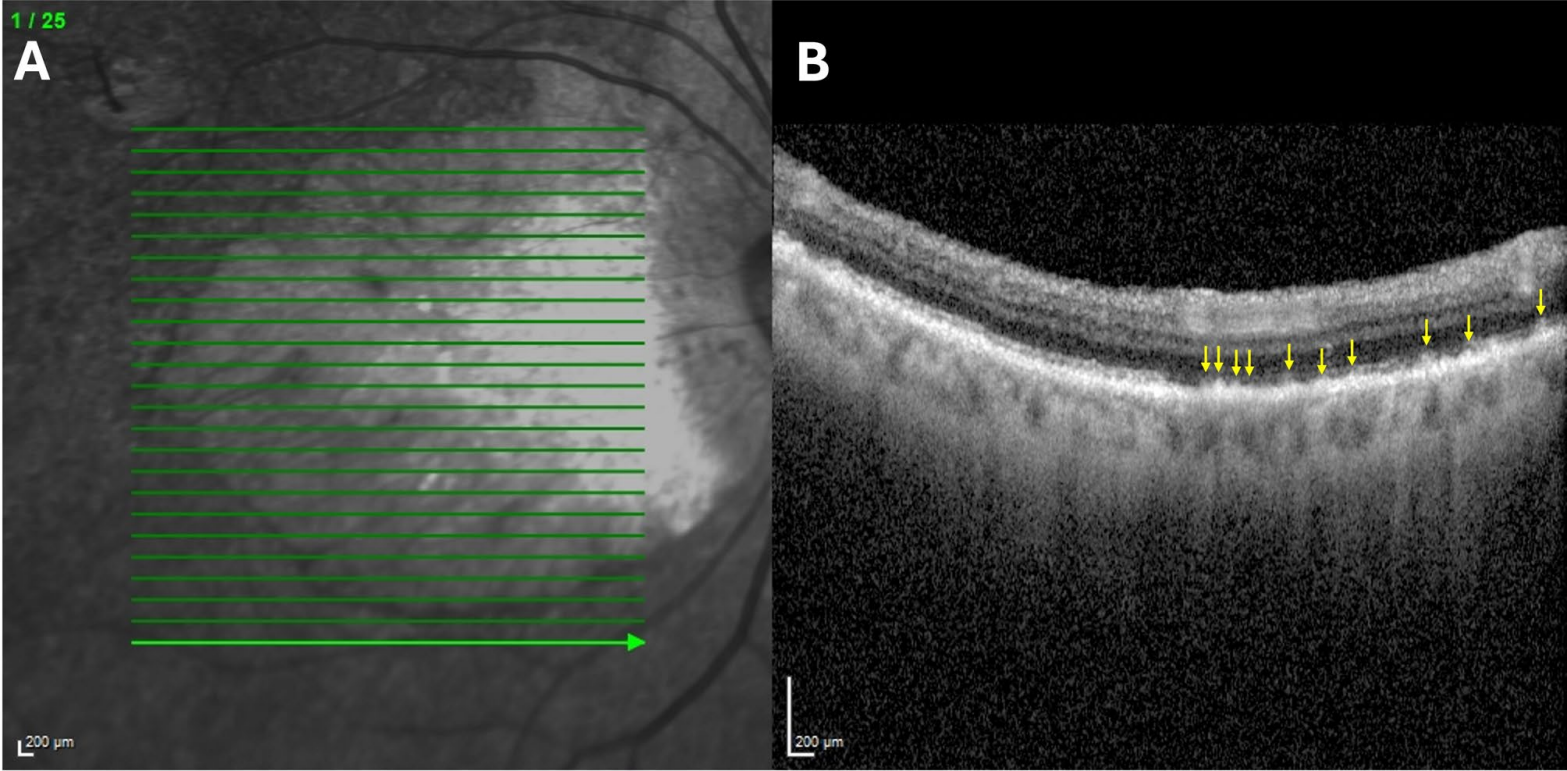
Black subject with central geographic atrophy and cuticular drusen, right eye. (**A**) Near-infrared reflectance (NIR) scan. There is extensive peripapillary and RPE atrophy. The green line indicates the location of SD-OCT scan which passes through a non-atrophic area. (**B**) Spectral domain optical coherence tomography (SD-OCT) B-scan. The B-scan reveals many saw tooth pattern elevations of the RPE in the sub-RPE-basal laminar compartment, characteristic of cuticular drusen (yellow arrows)

**Fig. 5:**
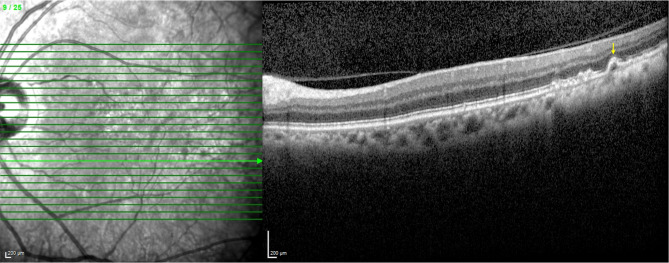
Hispanic subject with calcified drusen, left eye. (**A**) Near-infrared reflectance (NIR) scan. There are multiple hyperreflective drusen present. The green line indicates the location of SD-OCT scan. (**B**) Spectral domain optical coherence tomography (SD-OCT) B-scan. B-scan reveals calcified drusen with characteristic hyperreflective caps and partially hyporeflective cores (yellow arrows)

Figure 1 shows two different Hispanic subjects, one with just soft drusen and no SDDs and one with SDDs and no soft drusen. Neither of these subjects had HRVDs. Figures 2 and 3 are from subjects who had both SDDs and HRVDs. Figure 2 is from a Black subject with heart failure whereas Fig. 3 is from a Hispanic subject with aortic regurgitation. Figure 4 is from a Black subject with cuticular drusen. Figure 5 is from a Hispanic subject with calcified drusen.

## Discussion

To our knowledge, this is the first report of SDDs, cuticular drusen, and calcified drusen in Black or Hispanic AMD patients. Compared to soft drusen, SDDs, cuticular drusen, and calcified drusen are correlated with greater risk of development of advanced AMD and features of advanced AMD [5, 11, 12]. SDDs, specifically, are also associated with HRVDs [6]. Additionally, compared to Caucasians, Black and Hispanic populations have increased risk, morbidity, and mortality caused by the HRVDs [23–25], which, as noted, are associated with SDDs. A diligent search for SDDs, cuticular drusen, and calcified drusen in minority AMD patients may be warranted to assess the risk of progression to late AMD and to potentially recognize and treat HRVDs in minority SDD patients.

Cardiovascular disease screening, across a variety of screening tests, has been shown to be cost-effective for both the healthcare system and the patient [26, 27]. While the correlation between SDDs and HRVDs in Black and Hispanic populations needs to be studied further, the correlation between both diseases in the parent study combined with the increased risks associated with HRVDs in Black and Hispanic populations should encourage the potential implementation of and the acquisition of more robust data to support future screening protocols for SDDs and HRVDs. In fact, larger ophthalmic studies in Black and Hispanic populations have been done previously that demonstrate the feasibility for future studies to provide further support for SDD and HRVD screening in Black and Hispanic populations [28, 29]. While this study does not prove these screenings should be implemented, future screenings that are a result of more robust data can be implemented very similarly to diabetic retinopathy referrals for patients with existing diabetes. Cardiologists with patients who have HRVDs could refer them to ophthalmologists to identify SDDs. Ophthalmologists who discover SDDs in their patients could refer them to cardiologists for vascular workup.

Although statistical analysis was not done, due to a smaller sample size, key similarities were seen between our cohort of Black and Hispanic patients and patients from the larger parent study and literature. Importantly, the prevalence of SDDs in Black and Hispanic subjects (10/23, 43.5%) was relatively consistent with the total prevalence of SDDs in our original study (97/200, 48.5%) and in larger studies that have reported prevalences ranging from 9 to 70% with averages around 50% [3, 4, 6], As for Caucasian subjects specifically, our original study included 172 Caucasians, with an SDD prevalence of 86/172 (50.0%), similar to that of our small minority subgroups (10/23, 43.5%). Additionally, OCT imaging of SDDs (shown in Figs. 1, 2 and 3) revealed no differences between SDDs in our Black and Hispanic subjects and those in Caucasians in the literature. In these small groups, serum HDL and the choroid were also, respectively, lower and thinner in SDD subjects than drusen only subjects. These findings are consistent with those of the parent study of 200 subjects [6] that showed significantly lower HDL levels in patients (61 mg/dL when SDDs are present vs. 69 mg/dL when SDDs are not present) [6, 22], and with previous studies that have consistently found SDDs are associated with thinner choroids (between 30 and 60 μm thinner compared to AMD patients without SDDs) [30–32]. We recognize that the right and left eye choroidal measurements of our subjects are measured separately and thus may be subject to intra-subject correlation. This may cause the presented values to be a less accurate measurement, and as such they have not been utilized for statistical analysis but solely for informational comparison to choroidal thickness trends in literature. To our knowledge, choroidal thickness is not associated with cuticular or calcified drusen in literature and thus was not analyzed.

OCT imaging in cuticular and calcified drusen (Figs. 4 and 5) also showed no differences between these drusen subtypes in our Black and Hispanic subjects and those in Caucasians in the literature. Future studies with larger cohorts are needed to accurately analyze the prevalence of cuticular and calcified drusen in Black and Hispanic populations, but even within our smaller cohort, 17.4% of subjects had cuticular drusen and 17.4% of subjects had calcified drusen which is consistent with the prevalences of these drusen phenotypes in the general AMD population (approximately 25% and 20% respectively) [10, 33]

These data together suggest, but do not prove, that the SDD, cuticular drusen, and calcified drusen phenotypes in Black and Hispanic patients most likely have an epidemiology and physical structure similar to those in Caucasians. All these points require validation in larger groups.

The weakness of this study lies in any extrapolations to larger populations from the small numbers. The small numbers, of course, simply make it even more imperative to search specifically in the large, known minority populations with AMD [16] for the aforementioned SDDs, cuticular drusen, and calcified drusen phenotypes, which may well be prevalent. The strength of the study aims not to generalize pathologic findings or provide definitive follow-up protocols, but to elucidate this knowledge gap, emphasize the necessity of further work to fill it, and thus further advocate for populations that are already underserved by the healthcare system [34, 35]. Additionally, small population studies, even in AMD, have demonstrated value in understanding disease processes and bringing about positive downstream outcomes for patients [36]. The main point is the definite identification of SDDs, cuticular drusen, and calcified drusen in Black and Hispanic populations, which are new, important findings about AMD in minorities. Although AMD is less prevalent in Black and Hispanic populations, our findings are strengthened by the identification of similar phenotypes, morphology, and distribution, of SDDs, cuticular drusen, and calcified drusen in Black and Hispanic subjects who do have AMD when compared to Caucasian populations.

Future research should be focused on larger population studies comparing SDDs, cuticular drusen, and calcified drusen in Black and Hispanic AMD patients to White AMD patients. Similarly to the Salisbury Eye Evaluation Project [37], these studies can help reveal any differences that may be present between these populations, as well as provide more robust evidence and statistical comparisons for the presence, prevalence, and phenotypes of SDDs, cuticular drusen, and calcified drusen in Black and Hispanic populations.

## Conclusion

In summary, this study is the first to our knowledge to report subretinal drusenoid deposits, cuticular drusen, and calcified drusen in Black and Hispanic AMD subjects. In addition, pursuant to the recent discovery of the association of SDDs with high-risk vascular diseases [6], we feel that it is even more important to fill this longstanding knowledge gap about SDDs in minorities with AMD. It is important for the proper care of disadvantaged populations by eye specialists. Recognition of SDDs in Black and Hispanic patients can potentially help improve care for life-threatening cardiovascular diseases, which are overrepresented in these populations, and recognition that SDDs, cuticular drusen, and calcified drusen exist in Black and Hispanic patients can help improve identification and subsequent treatment of AMD.

## Electronic supplementary material

Below is the link to the electronic supplementary material.

Supplementary Material 1

## Data Availability

The datasets used and/or analysed during the current study are available from the corresponding author (RTS) on reasonable request.

## Abbreviations

AMD: Age-related macular degeneration
iAMD: Intermediate age-related macular degeneration
SDD: Subretinal drusenoid deposit
HRVD: High-risk vascular disease
RPD: Reticular pseudo drusen
SD-OCT: Spectral-domain optical coherence tomography
RPE: Retinal pigment epithelium
sfCTh: Sub-foveal choroidal thickness
MI: Myocardial infarction
CHF: Congestive heart failure
TIA: Transient ischemic attack
CVD: Cardiovascular disease
CABG: Coronary artery bypass graft
HDL: High-density lipoprotein
LDL: Low-density lipoprotein
