# Supplementary material for: Age-related macular degeneration, subretinal drusenoid deposits, and cuticular and calcified drusen in black and hispanic subjects
Source: Int J Retina Vitreous. 2025 Jul 28;11:85. doi: 10.1186/s40942-025-00710-4 (PMC12302460; doi:10.1186/s40942-025-00710-4)
Supplement: Supplementary file 1 — Supplementary Material 1 [file 40942_2025_710_MOESM1_ESM.docx]

**Additional File 1: Supplementary methods from the parent study**

The methods as follows are, in part, stated verbatim from the parent study, but listed here for reference.

The parent study was a cross-sectional study of two cohorts (SDDs and drusen only randomly selected AMD subjects for classification by retinal imaging). Slightly varying definitions of iAMD have been used; most commonly the defining lesions are soft drusen and/or SDD,^1,2^ which was adopted. Known demographic, clinical and social risks for HRVD were obtained from a questionnaire, and serum risks for HRVD were drawn. Vascular histories classified subjects into HRVD and non-HRVD for statistical association with SDDs and other risks. It was a cross-sectional study conducted at two tertiary vitreoretinal referral centers in New York City, New York, USA: Vitreous Retina Macula Consultants of New York (VRM) (LAY and KBF), and Department of Ophthalmology, New York Eye and Ear Infirmary (NYEE) of Mount Sinai School of Medicine (MSSM) (RBR and RTS), from August 2019 to November 2021, with 14-month interruption from COVID-19. The institutional review board (IRB) of MSSM approved the study, IRB approval no 19-00437, which adhered to the tenets of the Declaration of Helsinki.

**Inclusion criteria**

Subjects were aged 51–100 years, diagnosed with iAMD in at least one eye. All subjects had the capacity to sign informed consent and complete a study-related questionnaire. However, selection bias against more infirm or less able to cooperate subjects was discouraged.

**Exclusion criteria**

Bilateral advanced AMD. Other retinal degenerations and retinal vascular diseases such as diabetic retinopathy, prior retinal surgery (except intravitreal injections) and/or inconclusive medical or macular diagnoses.

**Demographic, clinical and social risk variables**

A questionnaire covered age, gender, race/ethnicity (white/black/Hispanic/Asian), body mass index (calculated), smoking history >6 months, treated hypertension or diabetes, use of blood thinners or antilipid medications.

**Vascular history for assignment to HRVD**

Subjects who reported stroke/TIA, MI, CABG, angina, arrhythmia, positive stress test, positive cardiac catheterization, stent, valve disease and CHF were the CVD and stroke group. Among them, HRVD status was assigned to two cardiovascular categories: myocardial defects (such as MI, CHF) and valve defects; and one neurovascular, stroke/TIA. These categories were determined by experts in the field (JN, MSD) as likely causes of decreased systemic or ocular perfusion. All other subjects were classified as non-HRVD. The HRVD cases were checked against medical records; valve disease was further reviewed for specific valve(s), stenosis and/or insufficiency; strokes/TIA were reviewed for laterality.

**Ophthalmic history, examination and multimodal imaging**

Historical variables were age of AMD onset, AMD in first-degree relatives and intravitreal injections either eye (Y/N).

All examinations and image classifications were performed uniformly by two authors (GL-G, OO-M), including slit-lamp examination for iris color (dark/light) and lens status (phakic/pseudophakic).

Volume SD-OCT scans (27 lines, automated retinal tracking, 16 scans averaged per line, good quality at least (29–34) per the device specifications), and en face AF and NIR scans (both 30°) centered on the macula, were obtained on the Heidelberg Spectralis HRA+OCT (Heidelberg Engineering, Heidelberg, Delaware, USA). Subjects without good quality SD-OCT scans were removed. After enrolment, SD-OCT images were analyzed for SDD following a published protocol.^3^ In the case of unilateral nAMD, all determinations were made on the fellow eye. AF and NIR images were used to confirm the presence of SDDs.^3^ Soft drusen were identified by standard criteria. Subjects were assigned to two groups: SDD (SDD either eye, ±drusen) and drusen (drusen only). Mean choroidal thickness was measured on central SD-OCT scans.

**Serum risks**

Blood samples for risk biomarkers of ASD^4^ (high-density lipoprotein (HDL), low-density lipoprotein (LDL), triglycerides) and high-sensitivity C reactive protein (hsCRP) were rapidly centrifuged at 1800*g* for 10 min and refrigerated. Fasting is no longer considered necessary for lipid levels,^5^ which were measured (Quest Diagnostics, Teterboro, New Jersey, USA) by spectrophotometry, and plasma levels of hsCRP by Immunoturbidimetric Assay (Orion Diagnostica, Finland).

**Power calculation, statistics and study outcomes**

Reported ORs for SDD versus drusen for any coronary artery disease (CAD) were 2:1,^6^ and for any AMD versus no AMD were also 2:1 for severe versus mild CAD.^7^ Hence, we estimated an OR of 4:1 for SDD versus drusen for severe versus mild CAD. Comparing 100 SDDs to 100 drusen, subjects yields power of 80% to detect an OR of at least 2.0 at alpha=0.05.

Univariate statistics for continuous variables with >30 samples were two-tailed t-tests and quartiles otherwise, and χ^2^ for categorical variables. Significant continuous variables were converted to quartile categories for multivariate testing, which then corrected the significance of each covariate for all others. Primary outcomes were thus 23 univariate and 1 standard multivariate correlation of SDD status (Y/N) and all 22 listed demographic, ocular, clinical and systemic risks with HRVD. Secondary outcomes were 23 univariate and 1 multivariate correlations of HRVD status and all 22 other risk variables with SDD status. Data scientists (AB and AG) used ‘IBM SPSS Statistics V.27’, ‘Waikato Environment for Knowledge Analysis (WEKA) V.3.8.5’, a data modelling tool and Microsoft Excel 365. Significance was set at p<0.05. Subjects with discordant iris color (none) or lens status (17) were removed from univariate statistics for these variables. Variables that retained multivariate significance p<0.05 were then entered into a standard linear regression model for HRVD risk, with coefficients that maximized the adjusted R-squared statistic. Final study outcomes were accuracy, specificity and sensitivity of the final model for HRVD risk with 95% CIs.

**References**

1. Tan R, Guymer RH, Luu CD. Subretinal drusenoid deposits and the loss of rod function in intermediate age-related macular degeneration. *Investigative Ophthalmology & Visual Science*. 2018;59(10):4154-4161.

2. Xu X, Liu X, Wang X, et al. Retinal pigment epithelium degeneration associated with subretinal drusenoid deposits in age-related macular degeneration. *American journal of ophthalmology*. 2017;175:87-98.

3. Smith RT, Sohrab MA, Busuioc M, Barile G. Reticular macular disease. *American journal of ophthalmology*. 2009;148(5):733-743. e2.

4. Yao H, Hou C, Liu W, Yi J, Su W, Hou Q. Associations of multiple serum biomarkers and the risk of cardiovascular disease in China. *BMC Cardiovascular Disorders*. 2020;20:1-11.

5. Langsted A, Freiberg JJ, Nordestgaard BG. Fasting and nonfasting lipid levels: influence of normal food intake on lipids, lipoproteins, apolipoproteins, and cardiovascular risk prediction. *Circulation*. 2008;118(20):2047-2056.

6. Cymerman RM, Skolnick AH, Cole WJ, Nabati C, Curcio CA, Smith RT. Coronary artery disease and reticular macular disease, a subphenotype of early age-related macular degeneration. *Current eye research*. 2016;41(11):1482-1488.

7. Wang SB, Mitchell P, Chiha J, et al. Severity of coronary artery disease is independently associated with the frequency of early age-related macular degeneration. *British Journal of Ophthalmology*. 2015;99(3):365-370.
